# The optimal degree of lateral wedge insoles for reducing knee joint load: a systematic review and meta-analysis

**DOI:** 10.1186/s40945-019-0068-1

**Published:** 2019-12-19

**Authors:** Vitor Ferreira, Rita Simões, Rui Soles Gonçalves, Leandro Machado, Paulo Roriz

**Affiliations:** 1 School of Health Sciences, ESSUA - School of Health, Edificio 30, University of Aveiro, 3810-193 Aveiro, Portugal; 2 Santa Casa da Misericórdia da Mealhada, Aveiro, Portugal; 3 Polytechnic Institute of Coimbra, Coimbra Health School, Coimbra, Portugal University of Coimbra, Centre for Health Studies and Research, Coimbra, Portugal; 4 CIF2D, LABIOMEP, Faculdade de Desporto da Universidade do Porto, Coimbra, Portugal; 5 CIDESD-ISMAI, INESC-TEC, LABIOMEP, Coimbra, Portugal

**Keywords:** Lateral wedge insoles, External knee adduction moment, Osteoarthritis, Knee, Meta-analysis

## Abstract

**Background:**

Lateral wedge insoles are traditionally used to reduce the adduction moment that crosses the knee during walking in people with medial knee osteoarthritis. However, the best degree to reduce knee joint load is not yet well established.

**Methods:**

Electronic databases were searched from their inception until May 2017. Included studies reported on the immediate biomechanical effects of different degrees of lateral wedge insoles during walking in people with knee osteoarthritis. The main measures of interest relating to the biomechanics were the first and second peak of external knee adduction moment and knee adduction angular impulse. For the comparison of the biomechanical effects of different degrees of insoles, the studies were divided in three subgroups: insoles with a degree higher than 0° and equal to or lower than 5°; insoles higher than 5° and equal to or lower than 9°; and insoles higher than 9°. Eligible studies were pooled using random-effects meta-analysis.

**Results:**

Fifteen studies with a total of 415 participants met all eligibility criteria and were included in the final review and meta-analysis. The overall effect suggests that lateral wedge insoles resulted in a statistically significant reduction in the first peak (standardized mean difference [SMD] –0.25; 95% confidence interval [CI] –0.36, − 0.13; *P* < 0.001), second peak (SMD –0.26 [95% CI –0.48, − 0.04]; *P* = 0.02) and knee adduction angular impulse (SMD –0.17 [95% CI –0.31, − 0.03]; P = 0.02). The test of subgroups found no statistically significant differences.

**Conclusion:**

Systematic review and meta-analysis suggests that lateral wedge insoles cause an overall slight reduction in the biomechanical parameters. Higher degrees do not show higher reductions than lower degrees. Prior analysis of biomechanical parameters may be a valid option for selecting the optimal angle of wedge that best fits in knee osteoarthritis patients with the lowest possible degree.

## Background

Knee osteoarthritis (OA) is one of the most common forms of arthritis and is a leading cause of disability in older adults [1, 2]. Joint loads during walking are implicated in the pathogenesis of knee OA [3]. The ground reaction force (GRF) vector and the corresponding position vector, relative to the knee joint center, contribute to quantify the knee joint reaction force and illustrate better how load is distributed across the knee compartments. In fact, the external knee adduction moment (EKAM), mainly used in the study of knee OA, is obtained from the cross product between the frontal plane components of the previous vectors. During walking, the forces across the knee are not transmitted equally between the medial and lateral compartments [4]. The medial compartment has a higher load prevalence in subjects with tibiofemoral OA relative to the lateral compartment, especially in men [5]. A reduction in the EKAM leads to a change in medial-to-lateral distribution and a relative lowering of the internal forces in the medial compartment [6].

Yasuda and Sasaki, in the 1980s, originally described the potential of lateral wedged insoles to manage medial knee OA [7]. Lateral wedge insoles are placed inside shoes and shift the point of application of the GRF toward the outside of the foot (laterally), reducing the moment arm (i.e., the position vector that is normal to the GRF vector) during walking [8]. Therefore, the magnitude of the EKAM is also reduced, leading not only to a redistribution of knee load but also to a reduction of the load at the medial compartment [9, 10]. However, a current meta-analysis demonstrated that lateral wedge insoles caused small reductions in the EKAM and knee adduction angular impulse (KAAI) during walking, which could be ineffective in people with medial knee OA [11]. Moreover, it is estimated that at least 20% of the individuals using lateral wedge insoles could even increase EKAM during gait [8, 12–15]. Nevertheless, it is reasonable to argue that some factors may have affected the previous outcomes, such as the type of insole and the wedge degree. Several variations of the insole have been described, ranging from a wedge only on the heel [16–18] to one on the whole foot [19–21] and with [22–24] or without arch support [25–27]. On the other hand, most of the studies have been performed using the same wedge degree for all individuals, typically a 5 or 6 wedge degree [13, 20, 28, 29]. This lack of customization of insoles has been proposed as a relevant research question in an Arnold’s Editorial Journal article, “One size fits all, some or none?” [30]. Certainly, it is a question that needs more research.

To our knowledge, no systematic review pursues an understanding of the effects of the amount of wedge insole angulation on biomechanical factors associated with medial knee OA. Reviews have sought to distinguish the effects on biomechanics, but only on the global effect of several types of angulations [11] or on the influence of an arch support in the wedge insoles [31]. Therefore, the main objective of the present review was to determine the biomechanical effects of lateral wedge insoles of different angles in people with knee OA and attempt to understand whether any angulation is more effective in improving biomechanical parameters in patients with knee OA.

## Methods

A systematic literature search was conducted, following the Preferred Reporting Items for Systematic Reviews and Meta-Analyses (PRISMA) group statement [32]. The study protocol is registered in PROSPERO (International prospective register of systematic reviews), with registration number CRD42017070785 (http://www.crd.york.ac.uk/prospero/).

The Population, Intervention, Comparison, and Outcome (PICO) framework was used to define the search strategy [33]: P (population) individuals diagnosed with knee osteoarthritis; I (intervention) that used lateral wedge insoles; C (comparison) who wore neutral insoles or their own shoes; O (outcome) first peak EKAM, second peak EKAM and KAAI when available.

Combinations of keywords and specific subject headings related to knee osteoarthritis, external knee adduction moment, biomechanics kinetics and kinematics, and interventions to reduce dynamic loading of the knee were employed.

Boolean operators “OR” and “AND” were used to combine search terms. No restrictions were set for the searches with respect to language or publication year. Two investigators (VF, RS) developed the search strategy (Table 1) and completed the systematic search. Medline/PubMed, CINAHL, and Scopus were searched from their inception through May 31, 2017. Syntax was adjusted appropriately for use in multiple databases. Keywords were identical for all searches. Keywords Medical Subject Headings (MeSH) proofed and non-MeSH proofed were used to increase the chances of finding the intended studies.

**Table 1:** Example of MEDLINE search strategy

Search	Query
1	knee
2	arthritis OR arthrosis OR osteoarthr*
3	1 AND 2
4	biomechanic* OR kinematics* OR kinetics*
5	3 AND 4
6	adduction moment
7	ekam* OR kam* OR varus moment* OR knee varus*
8	6 OR 7
9	lateral* OR wedge* OR insole*
10	5 AND 8
11	9 AND 10

### Eligibility criteria

The articles from those databases were collected, and duplicates were removed and cross-checked between the researchers to ensure agreement. Two authors (VF, RS) reviewed the titles and abstracts of all articles for eligibility based on the criteria list. When in doubt, full-text articles were reviewed. Disagreements were resolved by discussion until consensus was reached.

Studies to be included in this review must have been peer-reviewed studies that examined the acute biomechanical effects of laterally wedged insoles in patients with medial compartment knee OA, published as full text. There were no restrictions on design and severity of knee OA. Studies must have investigated a lateral wedged insole, defined as an in-shoe orthotic device with a degree of inclination toward the lateral border of the foot. No restrictions were made regarding the features of insoles (i.e., length: heel or full length) or presence of concurrent arch support in the device. Only baseline data inferring the immediate effects of lateral wedge insoles were used. Studies must have included a comparison condition that could be the insole removed or neutral insole (without any degree). If studies included the two control conditions (neutral insole or insole removed), only the data from neutral insole were analyzed. If an article analyzed customization intervention, but did not provide individual results, the article was excluded.

Systematic and narrative reviews were eligible for the purposes of a manual reference list, searching only to identify any studies missed in the primary search. Studies were excluded if they did not include patients with osteoarthritis or osteoarthrosis, and if they were abstracts, case reports, editorials, conference proceedings papers, study protocols or unpublished papers, or without full access.

### Data extraction

From the full articles that formed the results of this systematic review, the principal author extracted data of the characteristics of the individual trials and all outcomes into spreadsheets. A second author (RS) checked the data for accuracy. The main data extracted was the study design, number of patients, patient characteristics, type of insole, degree of lateral wedge, control condition, biomechanical outcome measures, statistical information, and funding sources. Primary outcome variables of interest extracted included features of the external knee adduction moment: the first peak EKAM, second peak EKAM, and KAAI.

### Assessment of risk of bias in included studies

The risk-of-bias assessment was completed by one author (VF) and checked by a second author (RS), using the Cochrane Risk of Bias assessment tool [34]. Each article was graded (unclear, low, or high risk of bias) based on selection bias (random sequence generation and allocation concealment), detection bias (blinding of outcome assessment), blinding of participants and personnel (performance bias), attrition bias (incomplete outcome data), reporting bias (selective reporting), and other bias. In case of disagreements, a common consensus was established and a third author (PR) was consulted if consensus could not be reached after consulting the guidelines of the software used.

### Data analysis

Statistical information, including descriptive (means, medians, standard deviations [SDs], change scores) and inferential (*P* values, confidence interval [CI]) information, was extracted and cross-checked by two authors (VF, RS). For the meta-analysis, standardized mean differences (SMDs) were calculated as the mean difference in EKAM change produced by the degree of the insole and the control (neutral insole or without insole), divided by the pooled standard deviation of the measurement. Hence, a negative effect size indicated a beneficial effect for the insole group. Effect sizes were interpreted as 0.2 (small), 0.5 (medium), and 0.8 (large) [35]. Meta-analysis was performed in Review Manager (RevMan) software (version 5.3, Cochrane Collaboration), using the inverse variance method [36], where the contribution of effect sizes from individual studies was weighted on sample size. For studies not reporting enough data, and where the authors could not provide data, they were calculated from other available data when possible (e.g., from 95% CI or *P* values from *t*-tests). It was decided to use a random-effects model, a priori, to estimate the pooled effect of intervention more conservatively.

Heterogeneity was assessed using a *χ*^2^ test (Q value), its corresponding degrees of freedom, and *p* value. The extent of heterogeneity was analyzed using Higgins’ *I*^2^ value (expressed as %) [37]. Heterogeneity determined the percentage of total variation across studies that is due to heterogeneity rather than to chance and examines the null hypothesis that all studies are evaluating the same effect [38]. For the interpretation of heterogeneity, the values of 25, 50, and 75% were followed, which represent low, moderate, and high heterogeneity, respectively [38]. The risk of small-study effects was assessed using the Egger’s regression test [34] and, if present, adjustment was planned using a trim-and-fill method [39] with STATA software (version 12, StataCorp).

## Results

### Study selection and characteristics

**Fig. 1:**
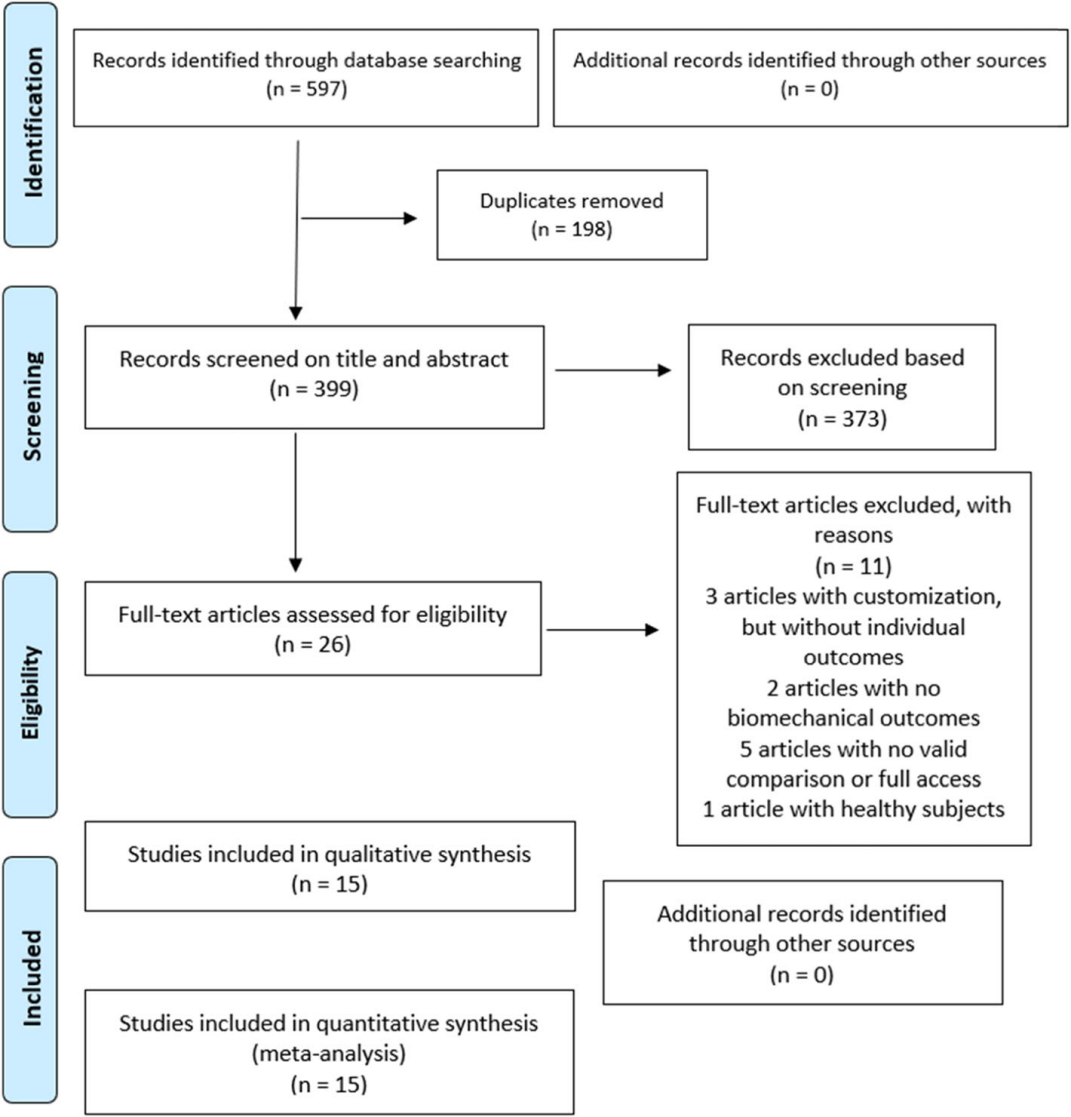
PRISMA flow diagram of the study selection process

A total of 597 records were identified, and 399 were screened on title and abstract. After assessing eligibility against the criteria, 26 studies were retained for full-text review. Fifteen studies, with a total of 415 participants, met all eligibility criteria and were included in the final review and meta-analysis (Fig. 1).

Values are indicated as mean ± SD unless indicated otherwise; M: F = male: female; (y) years; K/L = Kellgren/Lawrence; n/r – not reported.

Table 2 summarizes the characteristics of the included studies and participants. Seven studies had small samples sizes (*n* < 20) [9, 15, 18, 25, 40, 41, 44]. Most studies included participants aged over 45 years. The mean age was about the sixties. The principal criterion to classify OA severity was the Kellgren and Lawrence (K/L) scale [46]. Seven studies included only participants with grade 2 or 3 on the K/L scale [8, 13, 18, 20, 24, 25, 44, 45]. One study used an intervention with an insole of 4 degrees [44], eight studies used an intervention with an insole of 5 degrees [8, 9, 13, 18, 20, 40, 43, 45], five studies used an insole of 6 degrees [15, 24–26, 41, 42], two studies with an insole of 10 degrees [9, 25], and one study used an insole of 11 degrees [24]. A neutral insole was the comparison condition in four studies [9, 24, 25, 41]; nine studies used participants’ shoes [8, 15, 18, 20, 26, 40, 42–44], and two used control shoes [13, 45]. For the comparison of the biomechanical effects of different degrees of insoles, the studies were divided into three subgroups: comparison of interventions with insoles with a degree higher than 0° and equal to or lower than 5° (insole ≤5°); comparison of interventions with insoles higher than 5° and equal to or lower than 9° (insole > 5° and ≤ 9°); and comparisons of interventions with insoles higher than 9° (insole > 9°). These intervals were chosen because they have been the most studied in the literature.

**Table 2:** Characteristics of included studies (*n* = 15)

Authors (year)	n	Sex, M:F	Age (y)	Varus alignment (°)	K/L Grade severity (n)				Funding Source
					1	2	3	4	
Kerrigam et al. [9] (2002)	15	8:7	69.7 ± 7.6	n/r	0	0	10	5	Supported by the Ellison Foundation and by the US Public Health Service.
Maly et al. [40] (2002)	12	9:3	60 ± 9.39	6.67 ± 4.2	n/r	n/r	n/r	n/r	Drummond Foundation and the Natural Sciences and Engineering Research Council of Canada.
Kakihana et al. [41] (2005)	13	n/r	63.3 ± 5.6	2.5 ± 3.9	n/r	n/r	n/r	n/r	Not stated
Shimada et al. [42] (2006)	23	6:17	67.0 ± 8.7	6.2 ± 4.4	11	11	13	11	Not stated
Hinman et al. [43] (2008a)	40	16:24	64.7 ± 9.4	5.5°	3	10	11	16	University Grants and the Arthritis Foundation of Australia.
Hinman et al. [18] (2008b)	13	6:7	59.7 ± 6.2	1.9° ± 2.9	0	7	6	0	National Health and Medical Research Council of Australia
Hinman et al. [20] (2009)	20	8:12	63.5 ± 9.4	n/r	0	8	12	0	National Health & Medical Research Council of Australia and the ANZ Charitable Trusts
Abdallah and Radwan [24] (2011)	21	0:21	54.1 ± 7.4	176–180°	0	n/r	n/r	0	Not stated
Hinman et al. [8] (2012)	73	28:45	63.3 ± 8.4	0.9° valgus	0	41	32	0	National Health & Medical Research Council of Australia
Pagani et al. [44] (2012)	10	2:8	57.5 ± 7.1	2.1° ± 1.2	0	6	4	0	Institute of Biomechanics of the German Sport University Cologne.
Jones et al. [13] (2014)	70	43:27	60.3 ± 9.6	n/r	0	17	25	0	Arthritis Research UK and National Institute for Health Research Biomedical Research Unit Funding Scheme.
Duivenvoorden et al. [26] (2015)	42	14:28	54.0 ± 7.0	n/r	15	8	18	1	Not stated
Hatfield et al. [45] (2016)	26	4:22	64.0 ± 8.0	n/r	0	16	10	0	Pedorthic Foundation of Canada. Canadian Institutes of Health Research the Natural Sciences and Engineering Research Council of Canada, and the Michael Smith Foundation for Health Research
Dessery et al. [25] (2016)	18	8:10	54.5 ± 8.6	4.5° ± 2.8	0	15	3	0	Fonds de Recherche du Québec – Nature et Technologies. Natural Sciences and Engineering Research Council of Canada. Ergoresearch Inc
Lewinson et al. [15] (2016)	19	6:13	59.9 ± 7.4	n/r	5	2	3	9	Canadian Institutes of Health Research. Alberta Innovates Health Solutions. Killam Trusts. New Balance Athletic Shoe Inc

### Risk of bias

Inter-rater agreement for each item of the methodological quality assessment was moderate to high (k = 0.72 to 0.91). In 71% of trials, the random sequence generation had adequate or unclear risk of bias. Adequate allocation concealment was observed as low risk of bias in 19%, unclear in 52%, and high risk of bias in 29% of the included trials. Most of the studies were not blinded to the participants, personnel, or the outcome assessment, but the review authors’ judgement remained that the outcome was not likely to be influenced by lack of blinding because, in this type of evaluation, the data processing was carried out later. Therefore, the performance and the detection bias were considered 100% adequate. Incomplete outcome data were presented in 5% of the trials, and selective reporting was observed as low risk of bias in 86% of the trials (**see** Additional files 1 and 2).

SMD: standardized mean differences; Nm/kg: Newton-meter / kilogram; %BW*Ht: % body weight x height; mm: millimeter.

### Meta-analysis

**Fig. 2:**
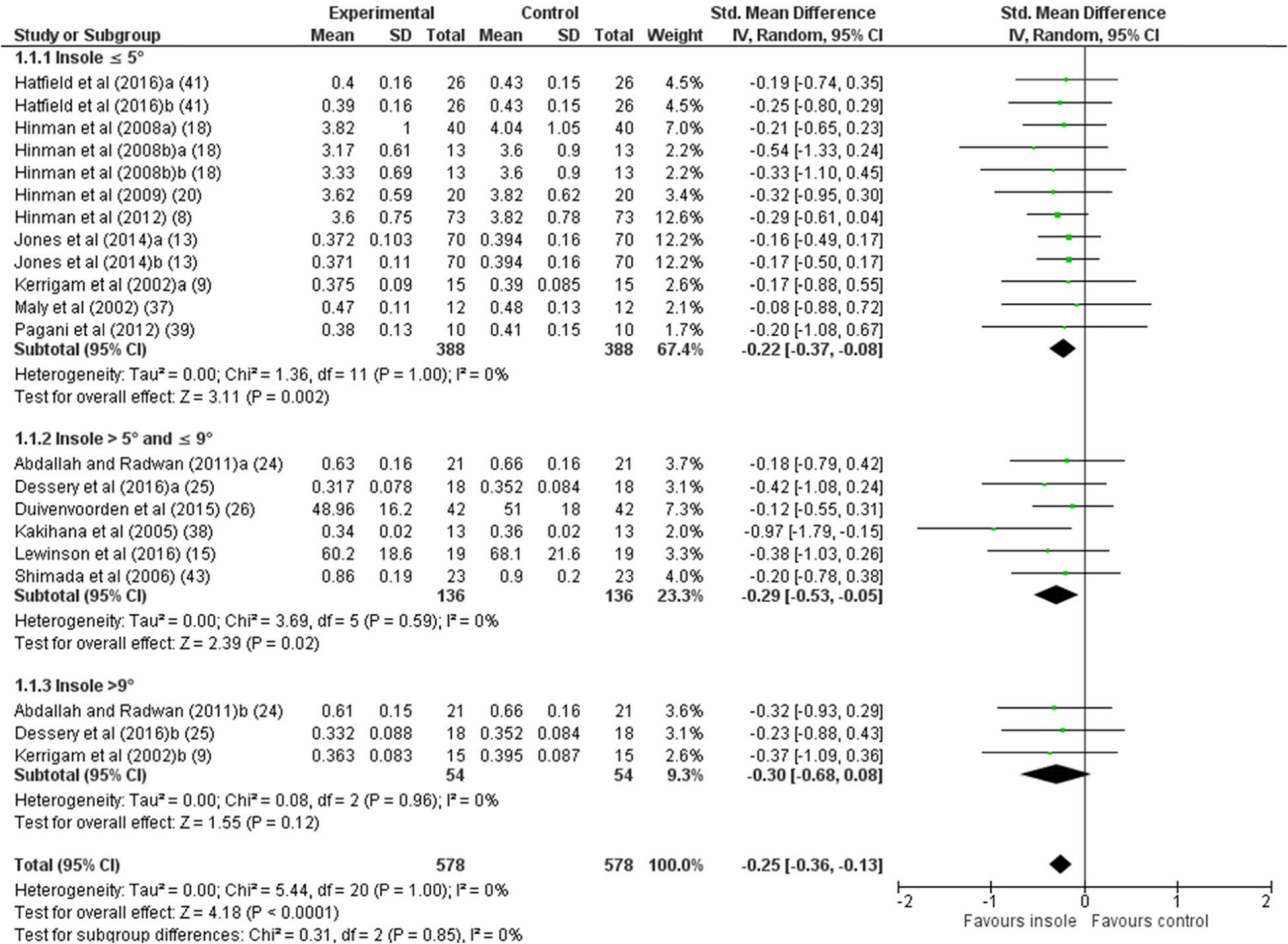
Forest plot of comparison: first peak external knee adduction moment (EKAM)

The **first peak EKAM** was the major outcome reported in the studies. All 15 studies stated the first peak EKAM. Because some studies made multiple comparisons with different features of the insole, such as the arch support or the length of the wedge, a total of 21 comparisons were included in the meta-analysis for the first peak EKAM (see Table 3). The overall effect suggests that lateral wedge insoles resulted in a statistically significant reduction in the first peak EKAM (*n* = 578, SMD –0.25 [95% CI –0.36, − 0.13], *P* < 0.001), with a low level of statistical heterogeneity (*x*^2^ = 5.44, *P* = 1.00, *I*^2^ 0%) (Fig. 2).

**Table 3:** Comparisons of interventions of included studies

Authors (year)	Unit of measure	Comparisons		SMD (95% CI)
		Intervention	Control	
First peak EKAM				
Kerrigam et al. [9] (2002)a	Nm/Kg*m	5° insole	5° Control insole (3.175 mm)	−0.17 [− 0.88, 0.55]
Kerrigam et al. [9] (2002)b	Nm/Kg*m	10° insole	10° Control insole (6.35 mm)	− 0.37 [− 1.09, 0.36]
Maly et al. [40] (2002)	Nm/Kg	5°insole	Participant shoes	− 0.08 [− 0.88, 0.72]
Kakihana et al. [41] (2005)	Nm/Kg	6° insole	Neutral insole	− 0.97 [− 1.79, − 0.15]
Shimada et al. [42] (2006)	Nm/Kg	6° insole	Participant shoes	− 0.20 [− 0.78, 0.38]
Hinman et al.) [43] (2008a	%BW*Ht	5° insole	Participant shoes	−0.21 [− 0.65, 0.23]
Hinman et al. [18] (2008b)a	%BW*Ht	5° full-length wedges	Participant shoes	− 0.54 [− 1.33, 0.24]
Hinman et al. [18] (2008b)b	%BW*Ht	5° rearfoot wedges	Participant shoes	− 0.33 [− 1.10, 0.45]
Hinman et al. [20] (2009)	%BW*Ht	5° insole	Participant shoes	−0.33 [− 1.10, 0.45]
Abdallah and Radwan [24] (2011)a	Nm/Kg	6° insole	Neutral insole	− 0.18 [− 0.79, 0.42]
Abdallah and Radwan [24] (2011)b	Nm/Kg	11° insole	Neutral insole	− 0.32 [− 0.93, 0.29]
Hinman et al. [8] (2012)	%BW*Ht	5° insole	Participant shoes	− 0.29 [− 0.61, 0.04]
Pagani et al. [44] (2012)	Nm/Kg	4° insole	Participant shoes	−0.20 [− 1.08, 0.67]
Jones et al. [13] (2014)a	Nm/Kg	5° insole with arch support	Control shoes	−0.16 [− 0.49, 0.17]
Jones et al. [13] (2014)b	Nm/Kg	5° insole without arch support	Control shoes	− 0.17 [− 0.50, 0.17]
Duivenvoorden et al. [26] (2015)	Nm/kg	6° insole	Participant shoes	−0.12 [− 0.55, 0.31]
Hatfield et al. [45] (2016)a	Nm/kg	5° insole with arch support	Control shoes	−0.19 [− 0.74, 0.35]
Hatfield et al. [45] (2016)b	Nm/kg	5° insole without arch support	Control shoes	−0.25 [− 0.80, 0.29]
Dessery et al. [25] (2016)a	%BW*Ht	6° insole with arch support	Control insole	− 0.42 [− 1.08, 0.24]
Dessery et al. [25] (2016)b	%BW*Ht	10° insole with arch support	Control insole	− 0.23 [− 0.88, 0.43]
Lewinson et al. [15] (2016)	Nm	6° insole	Participant shoes	− 0.38 [− 1.03, 0.26]
Second peak EKAM				
Kerrigam et al. [9] (2002)a	Nm/Kg*m	5° insole	5° Control insole (3.175 mm)	−0.17 [− 0.89, 0.55]
Kerrigam et al. [9] (2002)b	Nm/Kg*m	10° insole	10° Control insole (6.35 mm)	−0.30 [− 1.02, 0.42]
Hinman et al. [43] (2008a)	%BW*Ht	5° insole	Participant shoes	−0.31 [− 0.75, 0.13]
Hinman et al. [18] (2008b)a	%BW*Ht	5° full-length wedges	Participant shoes	−0.34 [− 1.12, 0.43]
Hinman et al. [18] (2008b)b	%BW*Ht	5° rearfoot wedges	Participant shoes	− 0.17 [− 0.94, 0.60]
Hinman et al. [20] (2009)	%BW*Ht	5° insole	Participant shoes	−0.16 [− 0.78, 0.46]
Pagani et al. [44] (2012)	Nm/Kg	4° insole	Participant shoes	−0.18 [− 1.06, 0.70]
Dessery et al. [25] (2016)a	%BW*Ht	6° insole with arch support	Control insole	−0.27 [− 0.92, 0.39]
Dessery et al. [25] (2016)b	%BW*Ht	10° insole with arch support	Control insole	−0.33 [− 0.99, 0.33]
Knee adduction angular impulse				
Hinman et al. [20] (2009)	%BW*Ht	5° insole	Participant shoes	−0.14 [− 0.76, 0.48]
Hinman et al. [8] (2012)	%BW*Ht	5° insole	Participant shoes	−0.21 [− 0.54, 0.11]
Pagani et al. [44] (2012)	Nm/Kg	4° insole	Participant shoes	−0.15 [− 1.03, 0.73]
Jones et al. [13] (2014)a	Nm/Kg	5° insole with arch support	Control shoes	−0.14 [− 0.47, 0.19]
Jones et al. [13] (2014)b	Nm/Kg	5° insole without arch support	Control shoes	−0.19 [− 0.52, 0.15]
Duivenvoorden et al. [26] (2015)	Nm/kg	6° insole	Participant shoes	−0.02 [− 0.45, 0.40]
Hatfield et al. [45] (2016)a	Nm/kg	5° insole with arch support	Control shoes	−0.12 [− 0.66, 0.43]
Hatfield et al. [45] (2016)b	Nm/kg	5° insole without arch support	Control shoes	−0.12 [− 0.66, 0.43]
Dessery et al. [25] (2016)a	%BW*Ht	6° insole with arch support	Control insole	−0.34 [− 1.00, 0.32]
Dessery et al. [25] (2016)b	%BW*Ht	10° insole with arch support	Control insole	− 0.34 [− 1.00, 0.32]
Lewinson et al. [15] (2016)	Nms	6° insole	Participant shoes	−0.23 [− 0.87, 0.41]

The comparison for subgroups did not show a statistically significant difference (*x*^2^ = 0.31, *P* = 0.85, *I*^2^ 0%). Two subgroups presented statistically significant reductions: insole ≤5° (SMD –0.22 [95% CI –0.37, − 0.08], *P* = 0.002) and insole > 5° and ≤ 9° (SMD –0.29 [95% CI –0.53, − 0.05], *P* = 0.02). However, the subgroup insole > 9° showed no statistically significant reduction (SMD –0.30 [95% CI –0.68, 0.08], *P* = 0.12). The Egger’s regression test for funnel plot asymmetry was positive (β = − 0.75, standard error (SE) 0.33, *P* = 0.034), indicating weak evidence of publication bias for the first peak EKAM (see Additional file 3). When using the trim and fill method, no trimming was performed, and the data remained unchanged.

**Fig. 3:**
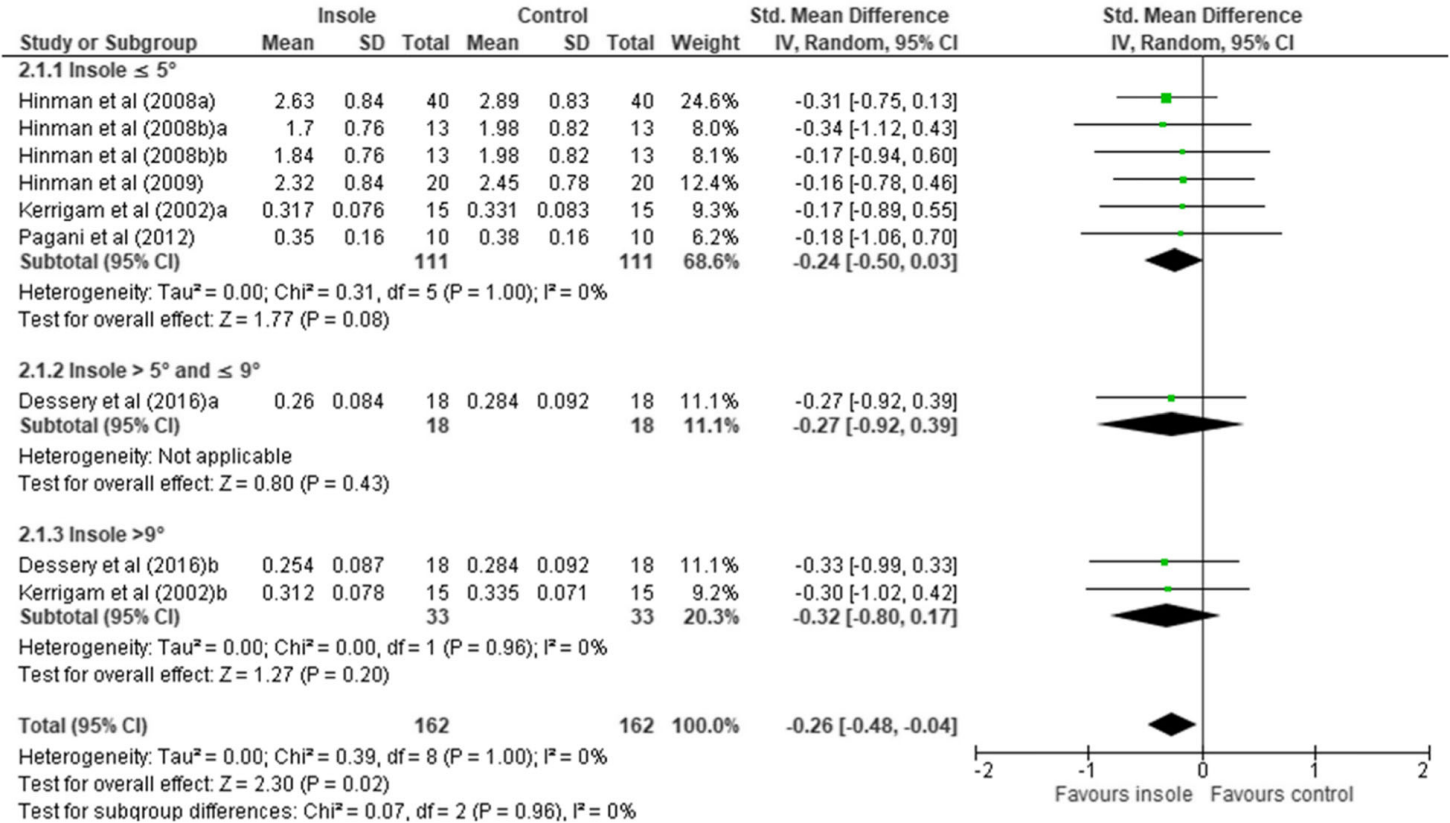
Forest plot of comparison: second peak external knee adduction moment (EKAM)

Only six studies reported the **second peak EKAM** [9, 18, 20, 25, 43, 44]. A total of nine comparisons were included in the data synthesis (Table 3). Six comparisons were for insoles ≤5°. The overall effect suggests that lateral wedge insoles resulted in a statistically significant reduction in the second peak EKAM (*n* = 162, SMD –0.26 [95% CI –0.48, − 0.04], *P* = 0.02), with a low level of statistical heterogeneity (*x*^2^ = 0.39, *P* = 1.00, *I*^2^ 0%) (Fig. 3).

None of the subgroups showed a statistically significant reduction of the overall effect, with pooled effect similar between them when compared with the control condition: insole ≤5° (SMD –0.24 [95% CI –0.50, 0.03], *P* = 0.08); insole > 5° and ≤ 9° (SMD –0.27 [95% CI –0.92, 0.39], *P* = 0.43); and insole > 9° (SMD –0.32 [95% CI –0.80, 0.17], *P* = 0.2). Egger’s regression test for funnel plot asymmetry was not statistically significant, indicating weak evidence of publication bias for the second peak EKAM (β = 0.40, SE 0.36, *P* = 0.306) (**see** Additional file 4).

**Fig. 4:**
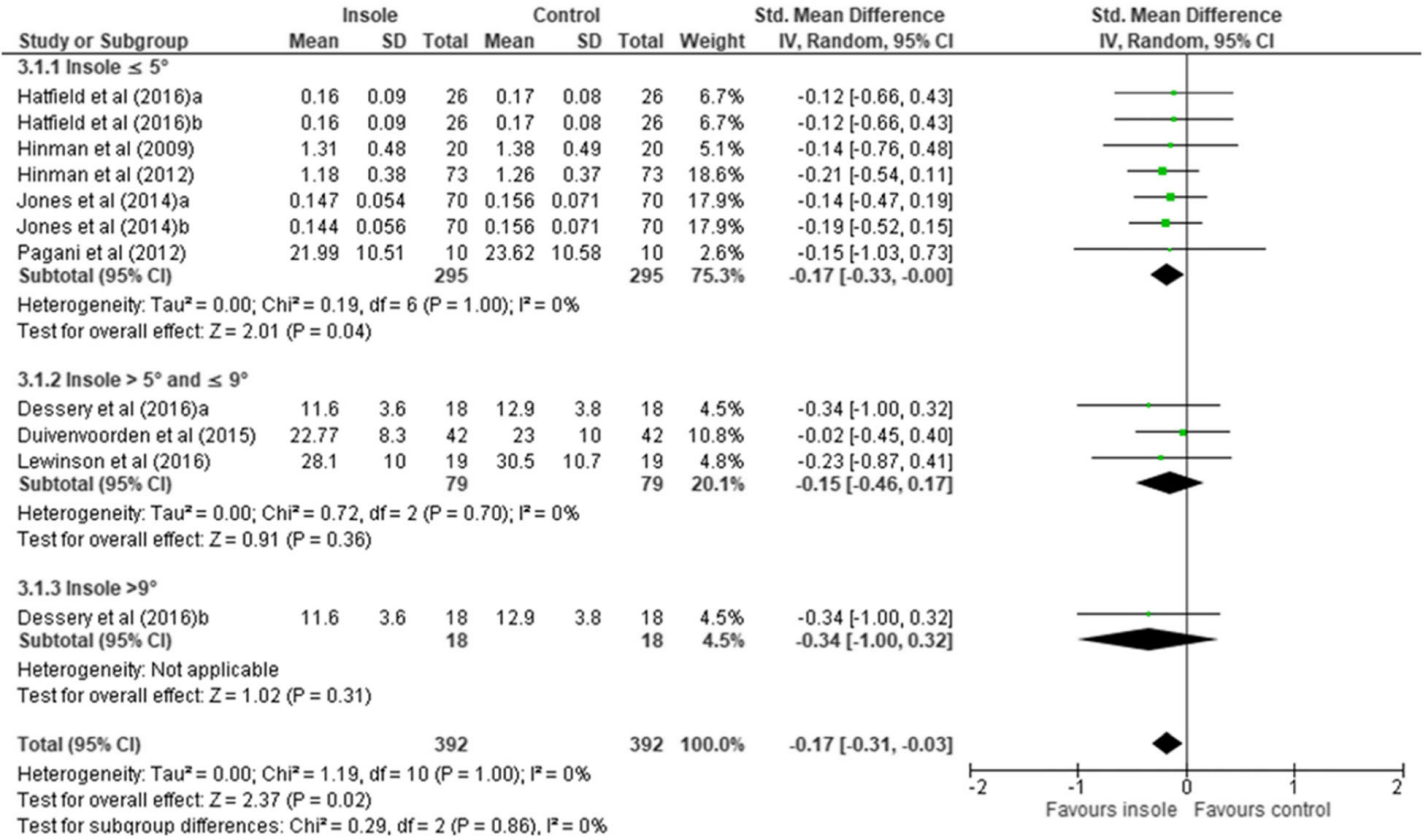
Forest plot of comparison: Knee adduction angular impulse (KAAI)

The **KAAI** was considered in the study of biomechanical risks in past years and was reported in eight studies [8, 13, 15, 20, 25, 26, 44, 45]. A total of 11 comparisons were included in the data synthesis (Table 3). The overall pooled estimate indicated that a statistically significant reduction in the KAAI favors lateral wedge insoles (*n* = 392, SMD –0.17 [95% CI –0.31, − 0.03], *P* = 0.02) with a low level of statistical heterogeneity (*x*^2^ = 1.19, *P* = 1.00, *I*^2^ 0%) (Fig. 4). Subgroup comparisons yielded different pooled effects (*x*^2^ = 0.29, *P* = 0.86, *I*^2^ 0%). The insole ≤5° showed association with KAAI compared to the control condition (SMD –0.17 [95% CI –0.33, − 0.00], *P* = 0.04). The subgroups insole > 5° and ≤ 9° (SMD –0.15 [95% CI –0.46, 0.17], *P* = 0.36) and insole > 9° (SMD –0.34 [95% CI –1.00, 0.32], *P* = 0.31) showed no associations with KAAI compared to the control condition. The Egger publication bias plot for funnel plot asymmetry was not statistically significant, indicating weak evidence of publication bias for the KAAI (β = − 0.21, SE 0.34, *P* = 0.559) (**see** Additional file 5).

## Discussion

The main objective of this review was to determine the biomechanical effects of different angles of lateral wedge insoles in people with knee OA and understand if different amounts of angulations induce different responses. This meta-analysis confirms that lateral wedge insoles cause an immediate reduction on knee load in conservative treatment for people with medial knee OA. Biomechanical parameters related to the medial knee load, including first peak EKAM, second peak EKAM, and KAAI were reduced with the use of a lateral wedge insole, apart from the amount of the degree. To the best of our knowledge, no previous reviews evaluated the effect of different angulations on biomechanical parameters. The latest meta-analysis [11, 31] regarding these issues did not focus on the effects of different angulations on reducing knee load in people with OA. In the review by Arnold et al. [11], the authors explore the overall effects of lateral wedge insoles on biomechanical risk factors and, in the subgroups analysis, studied the immediate effects among the application of lateral wedge insoles, neutral insoles, or shoes without insoles. In an up-to-date meta-analysis, Xing et al. [31] also studied the immediate effects of lateral wedge insoles, but in the subgroups analysis the authors studied the influence of arch supports on lateral wedge insoles compared to control shoes or neutral insoles. In these two meta-analyses, a small SMD was verified in the reduction of the first and second peak of EKAM and in KAAI. For the first peak EKAM, was found an SMD = − 0.19 ([95% CI –0.23, − 0.15], *P* < 0.001) in the meta-analysis of Arnold et al. [11], an SMD = − 0.22 ([95% CI –0.37, − 0.07], *P* = 0.001) in the meta-analysis of Xing et al. [31], and in the present meta-analysis a very similar SMD = − 0.25 ([95% CI –0.36, − 0.13], P < 0.001). With regard to the second peak EKAM, was found an SMD = − 0.25 ([95% CI –0.31, − 0.18], P < 0.001) in the meta-analysis of Arnold et al. [11], an SMD = − 0.26 ([95% CI –0.47, − 0.06], *P* = 0.01) in the meta-analysis of Xing et al. [31], and in the present meta-analysis an analogous value of SMD = − 0.26 ([95% CI –0.48, − 0.04], *P* = 0.02). Likewise, for the KAAI, was found an SMD = − 0.14 ([95% CI –0.21, − 0.07], P < 0.001) in the meta-analysis of Arnold et al. [11], an SMD = − 0.21 ([95% CI –0.39, − 0.02], P = 0.01) in the meta-analysis of Xing et al. [31], and in the present meta-analysis a similar value of SMD = − 0.17 ([95% CI –0.31, − 0.03], P = 0.02). All three meta-analysis support an immediate effect on the reduction of the adductor moment applied at the knee with the use of lateral wedge insoles. This positive effect may be independent of the presence of a lateral arch support [31], and the presence of a neutral insole as a comparator may not be totally inert [11]. Also, as present in this meta-analysis, the effect of a higher wedge degree does not seem to be very relevant compared to lower-angle insoles. In the same way, it should not be forgotten that insoles, particularly the ones with higher degrees, are associated with some discomfort with prolonged use [47].

The main objective of this review was to understand whether the amount of the angulation of the wedge influenced the EKAM and KAAI in patients with medial knee OA. It was our hypothesis that larger angulations would lead to a higher effect. However, the effect size of insoles with wedges ≤5° (SMD = − 0.22) and the effect size of insoles with wedges > 9° (SMD = − 0.30) are very similar for the first peak and for the second peak EKAM. For KAAI, because was retrieved only one study (*n* = 18) [25] that studied insoles with a wedge greater than 9°, it is not possible to form any conclusion. An emerging problem that would require further analysis is related to the correct adjustment of the insoles to each patient. Apparently, there is no research investigating an optimal dose–response concerning the degree of lateral wedge insoles for each patient based on biomechanical factors. From our knowledge, only one study attempted to examine the effect of incrementally increasing lateral wedge amounts on EKAM [47]. However, a key limitation of that study was that the participants were healthy and young. The authors tested seven inclinations of lateral wedging (0°, 2°, 4°, 6°, 8°, 10°, 12°). Yet, it is curious that with an insole angled at 2°, the average reduction was surprisingly 6.4% in the first peak EKAM and 5.1% in the KAAI, values that are similar when compared to studies with participants with medial knee OA, where insoles with angles of 5° and 6° are typically applied [8, 13, 26]. Some studies have attempted to apply lateral wedge insoles in a customization way but based on other indicators such as subjective comfort, pain relief, or static pedometer evaluation [16, 19, 48–50]. Their conclusions seem more promising than traditional applications based only on one degree for all individuals. In the study by Barrios et al. [48], the authors observed an increased EKAM over time (1 year) in the control group but not in the intervention group and, within the intervention group, the mechanical effectiveness of the lateral wedging did not decrease over time.

However, the extent of these effects remains ambiguous, with some authors suggesting that the effect size of the decrease in load on the medial compartment observed with lateral wedge insoles is too small to be clinically relevant such as reducing pain or symptoms [15, 27, 51]. On the other hand, other authors [22, 52] suggest that minor changes in knee load may have a positive effect on patients’ symptoms given the number of steps taken per day (about 6500) [51]. An interesting question that has not yet been answered is whether a difference of 1 or 2 degrees in the wedge could have an impact on the biomechanical parameters and, in particular, on the patient’s complaints over a long time, especially when referring to a chronic and evolutive disease with a great impact on patients’ health-related quality of life [53]. We know that the biomechanical response to insoles by patients with similar characteristics presents a considerable variability.

Some limitations can be addressed to the present review, primarily the heterogeneity between the study designs and the participants. Most of the studies were single-group crossover and, from our judgment, presented an unclear or high risk of bias in the selection bias (random sequence generation and allocation concealment). These could be a problem because, in past years, the literature sought to identify different knee OA phenotypes [54, 55]. The selection of participants based on biomechanical criteria included in randomized controlled trials should be the way forward, based on the prescription of biomechanical response. Another limitation is the different methodologies used in the studies to calculate EKAM. It is not clear enough in some studies how EKAM was calculated in the procedures, which may limit the comparison between studies. On the other hand, the different setup of placement of the passive markers can make comparison of the results difficult in an area that is so sensitive to small differences. Possible differences in sample size or differences in different gender elements can also be seen as a limitation in the comparison of results.

Setting the optimal angulation for each patient can contribute to improve outcomes in these patients. The results of this study may contribute to a better definition of individual angulation. Future studies should focus on optimizing the angulation of the insole and personalizing the intervention.

## Conclusion

This systematic review with meta-analysis suggests that lateral wedge insoles have a small effect on reducing the forces that cross the medial knee in people with medial knee OA, regardless of the angulation applied.

The path of customization of the interventions may be the right path, and the support of clinical biomechanics may play an important role in therapeutic decisions. The analysis of biomechanical parameters may be a beneficial option for the application of lateral wedge insoles for individuals with knee OA. The optimal degree should be obtained from individual fitting with the lowest possible angle that causes an important reduction of biomechanical risks.

Given the clear biomechanical benefits, further research is needed to investigate targeted use of lateral wedge insoles in biomechanical phenotypes over a longer time to determine conclusively the effects of lateral wedge insoles.

## Supplementary information

**Additional file 1.** Risk of bias graph: review authors’ judgements about each risk of bias item presented as percentages across all included studies.

**Additional file 2.** Risk of bias summary: review authors’ judgements about each risk of bias item for each included study.

**Additional file 3.** Funnel plot of comparison: first peak EKAM.)

**Additional file 4.** Funnel plot of comparison: second peak EKAM.

**Additional file 5.** Funnel plot of comparison: knee adduction angular impulse (KAAI).

## Data Availability

Please contact author for data requests.

## Abbreviations

CI: Confidence interval
EKAM: external knee adduction moment
GRF: Ground reaction force
K/L: Kellgren & Lawrence scale;
KAAI: Knee adduction angular impulse
OA: Osteoarthritis
SD: Standard deviations
SMD: Standardized mean differences
