# Supplementary material for: The optimal degree of lateral wedge insoles for reducing knee joint load: a systematic review and meta-analysis
Source: Arch Physiother. 2019 Dec 19;9:18. doi: 10.1186/s40945-019-0068-1 (PMC6921534; doi:10.1186/s40945-019-0068-1)
Supplement: Supplementary file 2 — Additional file 2. Risk of bias summary: review authors’ judgements about each risk of bias item for each included study. [file 40945_2019_68_MOESM2_ESM.docx]

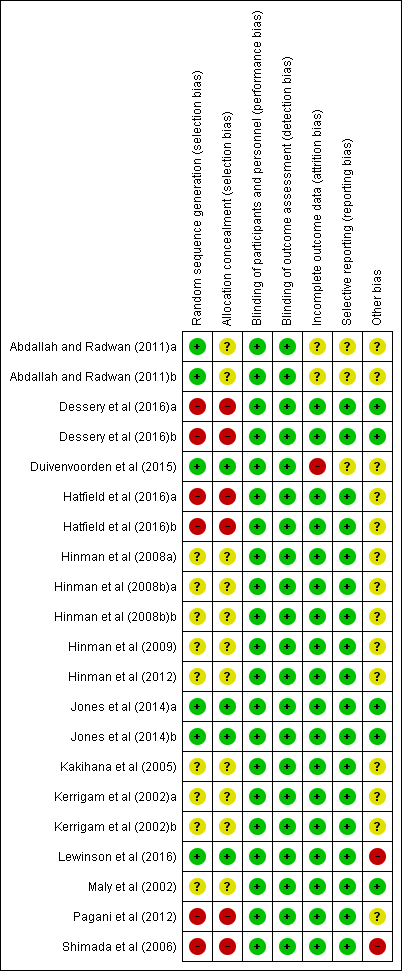


**Additional file 2**. Risk of bias summary: review authors' judgements about each risk of bias item for each included study: (+) – Low risk; (?) – Unclear risk; (-) – high risk.
