# Supplementary material for: The optimal degree of lateral wedge insoles for reducing knee joint load: a systematic review and meta-analysis
Source: Arch Physiother. 2019 Dec 19;9:18. doi: 10.1186/s40945-019-0068-1 (PMC6921534; doi:10.1186/s40945-019-0068-1)
Supplement: Supplementary file 3 — Additional file 3. Funnel plot of comparison: first peak EKAM.) [file 40945_2019_68_MOESM3_ESM.docx]

**Additional file 2.** Funnel plot of comparison: first peak EKAM
