# Supplementary material for: The optimal degree of lateral wedge insoles for reducing knee joint load: a systematic review and meta-analysis
Source: Arch Physiother. 2019 Dec 19;9:18. doi: 10.1186/s40945-019-0068-1 (PMC6921534; doi:10.1186/s40945-019-0068-1)
Supplement: Supplementary file 4 — Additional file 4. Funnel plot of comparison: second peak EKAM. [file 40945_2019_68_MOESM4_ESM.docx]

**Additional file 3**. Funnel plot of comparison: second peak EKAM
