# Supplementary material for: The optimal degree of lateral wedge insoles for reducing knee joint load: a systematic review and meta-analysis
Source: Arch Physiother. 2019 Dec 19;9:18. doi: 10.1186/s40945-019-0068-1 (PMC6921534; doi:10.1186/s40945-019-0068-1)
Supplement: Supplementary file 5 — Additional file 5. Funnel plot of comparison: knee adduction angular impulse (KAAI). [file 40945_2019_68_MOESM5_ESM.docx]

**Additional file 4**. Funnel plot of comparison: knee adduction angular impulse (KAAI)
